# Noncutaneous Infections in Patients with Hidradenitis Suppurativa: A Retrospective Cohort Study

**DOI:** 10.1016/j.xjidi.2025.100349

**Published:** 2025-01-21

**Authors:** Bruna G.O. Wafae, Alexandra P. Charrow, Megan H. Noe

**Affiliations:** 1 Department of Dermatology, Brigham and Women’s Hospital, Boston, Massachusetts, USA; 2 Harvard Medical School, Boston, Massachusetts, USA; 3 Beth Israel Deaconess Medical Center, Boston, Massachusetts, USA; 4 Sanofi Pharmaceuticals, Boston, Massachusetts, USA

**Keywords:** Acne inversa, Hidradenitis suppurativa, Hospitalization, Infection, Inpatient

## Abstract

Adults with hidradenitis suppurativa (HS) have comorbidities and are exposed to treatments that may increase their risk of serious infections. Therefore, our study aims to determine the prevalence and risk factors for noncutaneous infections (NCIs) related hospitalizations in adults with HS and analyze their patterns of healthcare utilization. This retrospective cohort included patients with dermatologist-confirmed HS from a single healthcare system between 2018 and 2022. Primary/secondary diagnostic codes identified NCI-related hospitalizations. Multivariable logistic regression assessed risk factors. Data on nonpsychiatric hospitalizations and emergency department visits were collected for overall healthcare utilization. Among the 834 patients with HS, 6.4% were hospitalized for NCI during the study period. The most common infections were urinary tract infections (18.2%), musculoskeletal infections (13%), and COVID-19 (11.7%). The main factors associated with NCIs were public insurance (OR = 2.06, confidence interval = 1.09–3.83), chronic kidney disease (OR = 7.73, confidence interval = 2.03–29.09), and anxiety (OR = 3.27, confidence interval = 1.58–6.67). Prevalence of nonpsychiatric hospitalization was 24.6%, and that of emergency department visits was 45.3%. In conclusion, patients with HS had a significant prevalence of hospitalizations from NCIs, with urinary tract infections being the most prevalent. The risk was higher in patients with anxiety or chronic kidney disease. Future research should focus on interventions and measures to prevent infections.

## Introduction

Current literature indicates that patients with hidradenitis suppurativa (HS) experience higher hospitalization rates than those with psoriasis and the general population (Kirby et al, 2014). Common HS treatments, including immunosuppressants and biological agents, are linked to increased infection risks (Chiu and Chen, 2020). Comorbidities in HS may further elevate the infection risk (Kimball et al, 2018). However, existing studies exploring serious infection in HS are limited by the cross-sectional design of inpatient data, misclassification bias, and lack of patient-specific factors. These studies may also have inflated infection rates owing to misclassification between cutaneous infections and HS flares, which is especially challenging for nondermatologists in acute settings. Therefore, our study aimed to determine the prevalence, types of infections, and clinical characteristics associated with hospitalizations due to noncutaneous infections (NCIs) in adults with HS and to understand patterns in healthcare utilization.

## Results

Of 834 patients with HS in our study, 53 individuals had 77 hospitalizations due to NCI, with a 5-year prevalence rate of 6.35%. These patients were mostly young, female, and White. Hospitalized and nonhospitalized groups were comparable in terms of demographic characteristics, smoking status, and body mass index. However, NCI-hospitalized patients were more likely to have certain comorbidities and to be covered under public insurance (Table 1). Systemic therapies were prescribed to 686 individuals (82.2%). The most common treatments included oral antibiotics (63.9%), hormone therapy (40.6%), and biologics (18%). In addition, 86 patients (10.3%) received oral corticosteroids. Hospitalized patients were more likely to receive antibiotics (moxifloxacin, metronidazole, and cephalexin) and infliximab. However, owing to the well-described limitations of treatment information on electronic medical records (Hersh et al, 2013), HS therapies were collected only for population descriptions.

**Table 1:** Demographic and Clinical Characteristics of Included Patients

Characteristics	Without Hospitalization from NCI Group (n = 781)	Noncutaneous Serious Infection Group (n = 53)	*P*-Value^1^
Age, median (IQR)	31.90 (25.50–41.70)	32.80 (27.90–49.10)	.139^2^
Sex, %			.788
Female	624 (79.9)	41 (77.4)	
Male	157 (20.1)	12 (22.6)	
Race, %			.616
Asian	44 (5.6)	3 (5.7)	
Black	214 (27.4)	19 (35.8)	
Other	89 (11.4)	7 (13.2)	
White	402 (51.5)	22 (41.5)	
Insurance, n (%)			**<.001**
Commercial/private	596 (76.3)	27 (50.9)	
Public	185 (23.7)	26 (49.1)	
Smoking status, n (%)^3^			.082
Never smoker	531 (67.9)	46 (88.5)	
Previous smoker	21 (2.7)	5 (9.6)	
Current smoker	27 (3.4)	<3^4^	
BMI, mean (SD)^3^	33.07 (8.10)	35.47 (10.21)	.052^2^
Pregnancy (%)	91 (11.7)	13 (24.5)	**.011**
Comorbidities, n (%)			
Anxiety	119 (15.2)	24 (45.3)	**<.001**
Asthma	99 (12.7)	17 (32.1)	**<.001**
Chronic kidney disease	7 (0.9)	7 (13.2)	**<.001**
Chronic obstructive Pulmonary disease	6 (0.8)	4 (7.5)	**.002**
Coronary heart disease	4 (0.5)	3 (5.7)	**.007**
Crohn’s disease	27 (3.5)	6 (11.3)	**.013**
Depression	154 (19.7)	22 (41.5)	**<.001**
Diabetes (type I and II)	44 (5.6)	9 (17.0)	**.003**
Dyslipidemia	77 (9.9)	8 (15.1)	.325
Hypertension	95 (12.2)	17 (32.1)	**<.001**
Ulcerative colitis	14 (1.8)	3 (5.7)	.088
Follow-up time, mo, mean (SD)	27.68 (17.93)	34.02 (17.62)	**.013**

Abbreviations: BMI, body mass index; IQR, interquartile range; NCI, noncutaneous infection.

Bold font indicates statistical significance.

1 P values were calculated with the use of the chi-square test, except where indicated.

2 P-value was calculated with Wilcoxon rank-sum test and t-test, respectively.

3 Missing data in 28.5% of participants for BMI and 25.8% for smoking status.

4 Count numbers were suppressed to preserve confidentiality.

The most common infections included urinary tract infection (18.2%), musculoskeletal infection (13%), COVID-19 (11.7%), upper respiratory infection, sepsis, liver/biliary tract infection, diverticulitis/appendicitis, chorioamnionitis (10.4% each), gastroenteritis/enterocolitis, peritonitis/gastrointestinal abscess, pancreatitis (5.2% each), pneumonia (3.9%), and CNS infection (<3%). A closer look at bloodstream infections (sepsis/bacteremia) showed that they were observed in 8 patients (10.4%). The primary infection sites for these infections were mainly cutaneous, with other infections, including urinary tract infection, osteomyelitis, gastrointestinal infection, and central line infection, also noted. All the observed infections are detailed in Table 2.

**Table 2:** Distribution of Noncutaneous Infections during the Study Period

Infection Site	Number of Infections (n = 77) n (%)
UTI infection and pyelonephritis	14 (18.2)
Musculoskeletal infection	10 (13)
COVID-19	9 (11.7)
Upper respiratory infection	8 (10.4)
Liver and biliary tract infections	8 (10.4)
Chorioamnionitis	8 (10.4)
Sepsis/bacteremia	8 (10.4)
Diverticulitis/appendicitis	8 (10.4)
Gastroenteritis or enterocolitis	4 (5.2)
Peritonitis and GI abscess	4 (5.2)
Pancreatitis	4 (5.2)
Lower respiratory tract infections, including pneumonia^1^	3 (3.9)
CNS infection	<3^2^

Abbreviations: GI, gastrointestinal; UTI, urinary tract infection.

1 Excluding pneumonia caused by COVID-19.

2 Count numbers were suppressed to preserve confidentiality.

In our risk factor analysis, we identified several independent risk factors for hospitalization: public insurance (OR = 2.06, 95% confidence interval = 1.09–3.83), anxiety (OR = 3.27, 95% confidence interval = 1.58–6.67), and chronic kidney disease (OR = 7.73, 95% confidence interval = 2.03–29.09), as shown in Table 3.

**Table 3:** Risk Factors for Hospitalization from Noncutaneous Infection

Predictors	OR^1^	95% CI
Age, y	1.00	0.97–1.02
Sex		
Female	Reference	—
Male	1.16	0.53–2.37
Healthcare insurance		
Commercial/private	Ref	—
Public	2.06	**1.09–3.83**
Crohn's disease	2.57	0.80–7.05
Asthma	1.61	0.76–3.27
Chronic obstructive pulmonary disease	4.53	0.81–22.55
Chronic kidney disease	7.73	**2.03–29.09**
Diabetes	1.17	0.38–3.07
Anxiety	3.27	**1.58–6.67**
Depression	0.88	0.40–1.86
Follow-up time, mo	1.02	1.00–1.04

Abbreviation: CI, confidence interval.

Bold font indicates statistical significance.

1 ORs were derived from multivariable logistic regression. A stepwise method and clinical relevance were used to select the predictors presented in the table.

With respect to overall healthcare utilization, 205 (24.6%) patients were hospitalized over the 5-year study period; of those, 39.5% were readmitted. Similarly, 378 (45.3%) patients visited the emergency department, and 69.8% had recurrent visits. NCIs accounted for 18.5% of all hospitalizations, and 30.2% were readmitted for NCIs.

## Discussion

In summary, 6.35% of adults with HS were hospitalized for NCIs, a notable figure given their young age and the relatively brief follow-up (34 months ± 17.6). The most frequent NCIs were urinary tract infections, musculoskeletal infections, and COVID-19, supporting previous findings regarding the impact of infections on HS (Lee et al, 2020).

Previous studies have reported conflicting results regarding the risk of HS infections. A cross-sectional study found a higher prevalence of infection in patients with HS than in the general population and in patients with psoriasis (Lee et al, 2020). Placebo-controlled trials of adalimumab and secukinumab had notably lower rates of infection and hospitalization across both the treatment and placebo arms; however, these were limited by short follow-up periods and restricted generalizability (Kimball et al, 2023, 2016).

Patients with NCIs had a higher prevalence of all the comorbidities examined, except for dyslipidemia and ulcerative colitis. In our analysis, chronic kidney disease was associated with NCI hospitalization. Although uncommon in HS, it is a significant risk factor for serious infections (Ishigami and Matsushita, 2019). We also identified anxiety as an independent risk factor for NCI, which may be mediated by its frequent co-occurrence with depression, a known infection risk factor (Andersson et al, 2016).

A substantial majority (82.2%) of patients were exposed to at least 1 systemic therapy for HS. Hospitalized patients showed a higher likelihood of receiving antibiotics (moxifloxacin, metronidazole, cephalexin) for treatment of HS as well as infliximab. Although standardized measures of disease severity were not available in this study, the medication profiles suggested that hospitalized patients were likely to have more severe HS on the basis of treatment patterns. Nevertheless, the effect of disease severity and/or medications on the risk of NCIs should be further explored to understand the role of each factor in the development of noncutaneous infections.

In addition, patients covered by public insurance were twice as likely to have NCI hospitalizations as those with private insurance. In our study, public insurance likely served as a proxy for the social determinants of health given the comprehensive insurance acceptance of the healthcare system studied. This finding resonates with prior studies (Lee et al, 2020) but warrants further investigation. Our findings also confirm the elevated hospitalization rates and emergency department visits previously reported in the literature (Edigin et al, 2021; Kirby et al, 2014).

The strengths of this study include the maximum of 5-year follow-up, individual characteristics, and clinical notes to confirm the diagnosis of HS and infectious outcomes. Generalizability may be limited owing to the urban academic medical center source, which may centralize complex cases. Moreover, this study focused on infections in an inpatient setting. Hospitalization rates may have been underestimated because of the lack of information on care outside our institution. The small sample size limits the power to evaluate all predictors and the magnitude of the association.

In conclusion, our study highlighted the prevalence and types of serious infections in adults with HS, emphasizing the need for education and monitoring of infections, particularly in patients with comorbidities. Our findings also demonstrate the reliance on inpatient and emergency department care by many patients with high readmission rates. Future studies should better characterize the pattern of infection in the outpatient setting as well as additional risk factors, including HS treatment, with larger, diverse samples and interventions to mitigate these risks.

## Materials and Methods

### Study design and study population

We conducted a retrospective cohort study of patients with HS in the Mass General Brigham system, approved by the institutional review board with consent exemption. Eligible patients included all adults (aged ≥18 years) with a dermatologist-confirmed HS diagnosis who received care in the Mass General Brigham system between January 1, 2018 and December 31, 2022. To ensure accuracy of HS diagnosis, we required at least 2 dermatology visits for HS to be eligible. Patients with a history of cancer (except nonmelanoma skin cancer), organ transplantation, or HIV were excluded.

### Outcome

The primary outcome was hospitalization due to NCI, identified through the principal, primary, and secondary diagnoses using validated and published International Classification of Diseases, Tenth Revision codes (Lo Re et al, 2021). We excluded cutaneous infections owing to the clinical overlap with HS flares and concern for misclassification. The secondary outcomes included infection type, nonpsychiatric hospitalizations, and emergency department visits. Given the study period, COVID-19 infections were analyzed separately from other respiratory infections.

### Predictors and covariates

The covariates of interest included sex, age, race, smoking status, body mass index, healthcare insurance (categorized as public for Medicare/Medicaid and private for other types), and comorbidities. Systemic therapies prescribed by a dermatologist during the HS visit were recorded as ever/never exposed.

### Statistical analyses

This study adhered to the Strengthening the Reporting of Observational Studies in Epidemiology reporting guidelines (von Elm et al, 2007). Statistical analyses were conducted using R Studio software, setting alpha values and confidence intervals at 0.05 and 95%, respectively.

The prevalence of hospitalization due to NCI was computed by dividing the number of patients hospitalized due to NCI by the total cohort. Infection types are described as frequencies with percentiles. Patient confidentiality was maintained by suppressing the counts to below 3.

Comparisons of categorical variables between patients with and without NCIs were performed using the chi-square and Fisher’s exact tests; the latter was used for small count numbers. Continuous variables were compared using *t*-tests for normally distributed variables and Wilcoxon rank-sum tests for non-normally distributed variables.

Risk factor analysis for the outcomes was dichotomized, and predictors were evaluated using a multivariable logistic regression model. The stepwise forward method selects health insurance type, Crohn's disease, asthma, chronic obstructive pulmonary disease, anxiety, and chronic kidney disease as the predictors in the model. Age, sex, and diabetes mellitus were included manually because of their clinical significance. To increase the accuracy of the model, all predictors were tested for collinearity, and all variables had a variance inflation factor below 2.0. Systemic HS therapies prescribed during the study period were not used as candidate predictors in the regression model because of the possibility of reverse causality, body mass index, and smoking owing to missing values (28.5 and 25.8%, respectively).

## Ethics Statement

This study was reviewed and approved by the Mass General Brigham Institutional Review Board (approval number 2022P002915). Patient consent was not applicable.

## Data Availability Statement

The data that support the findings of this study are available on request from the corresponding author. The data are not publicly available owing to privacy or ethical restrictions.

## ORCIDs

Bruna G. O. Wafae: http://orcid.org/0000-0003-4971-6029

Alexandra P. Charrow: http://orcid.org/0000-0002-3784-7091

Megan H. Noe: http://orcid.org/0000-0001-8481-4711

## Conflict of Interest

MHN received research grants from Boehringer Ingelheim and Bristol Myers Squib for research unrelated to this project. She worked as a consultant for Boehringer Ingelheim and Argenix and received honoraria. She is a senior editor of the *Journal of Psoriasis and Psoriasis Arthritis*. MHN is an employee of Sanofi. APC received funding from the Hidradenitis Foundation. She serves on an advisory board for UCB and Novartis and consulted for Q32. The remaining author states no conflict of interest.

## References

[bib1] Andersson N.W., Goodwin R.D., Okkels N., Gustafsson L.N., Taha F., Cole S.W. (2016). Depression and the risk of severe infections: prospective analyses on a nationwide representative sample. Int J Epidemiol.

[bib2] Chiu Y.M., Chen D.Y. (2020). Infection risk in patients undergoing treatment for inflammatory arthritis: non-biologics versus biologics. Expert Rev Clin Immunol.

[bib3] Edigin E., Kaul S., Eseaton P.O. (2021). Analysis of hidradenitis suppurativa hospitalizations: a report from the National Inpatient Sample database. J Am Acad Dermatol.

[bib4] Hersh W.R., Weiner M.G., Embi P.J., Logan J.R., Payne P.R.O., Bernstam E.V. (2013). Caveats for the use of operational electronic health record data in comparative effectiveness research. Med Care.

[bib5] Ishigami J., Matsushita K. (2019). Clinical epidemiology of infectious disease among patients with chronic kidney disease. Clin Exp Nephrol.

[bib6] Kimball A.B., Jemec G.B.E., Alavi A., Reguiai Z., Gottlieb A.B., Bechara F.G. (2023). Secukinumab in moderate-to-severe hidradenitis suppurativa (SUNSHINE and SUNRISE): week 16 and week 52 results of two identical, multicentre, randomised, placebo-controlled, double-blind phase 3 trials. Lancet.

[bib7] Kimball A.B., Okun M.M., Williams D.A., Gottlieb A.B., Papp K.A., Zouboulis C.C. (2016). Two Phase 3 trials of adalimumab for hidradenitis suppurativa. N Engl J Med.

[bib8] Kimball A.B., Sundaram M., Gauthier G., Guérin A., Pivneva I., Singh R. (2018). The comorbidity burden of hidradenitis suppurativa in the United States: a claims data analysis. Dermatol Ther (Heidelb).

[bib9] Kirby J.S., Miller J.J., Adams D.R., Leslie D. (2014). Health care utilization patterns and costs for patients with hidradenitis suppurativa. JAMA Dermatol.

[bib10] Lee H.H., Patel K.R., Singam V., Rastogi S., Silverberg J.I. (2020). Associations of cutaneous and extracutaneous infections with hidradenitis suppurativa in U.S. children and adults. Br J Dermatol.

[bib11] Lo Re V., Carbonari D.M., Jacob J., Short W.R., Leonard C.E., Lyons J.G. (2021). Validity of ICD-10-CM diagnoses to identify hospitalizations for serious infections among patients treated with biologic therapies. Pharmacoepidemiol Drug Saf.

[bib12] von Elm E., Altman D.G., Egger M., Pocock S.J., Gøtzsche P.C., Vandenbroucke J.P. (2007). The Strengthening the Reporting of Observational Studies in Epidemiology (STROBE) statement: guidelines for reporting observational studies. Lancet.

